# Odontogenic carcinosarcoma: a comprehensive review of clinical and therapeutic insights

**DOI:** 10.3389/froh.2025.1544921

**Published:** 2025-04-23

**Authors:** Muhammad Osama, Cyril Kocherry, Farid Ullah, Safiyyah Ubaid, Maryam Ubaid, Ubaid Ullah, Aishah Binte Nawaz, Hanan M. Qasem, Ramez M. Odat, Muzammil Farhan, Raheel Ahmed

**Affiliations:** ^1^ Internal Medicine, Khyber Medical College, Peshawar, Pakistan; ^2^ School of Medicine, University of Dundee, Dundee, United Kingdom; ^3^ Internal Medicine, Khyber Teaching Hospital, Peshawar, Pakistan; ^4^ Department of Biochemistry, Khyber Girls Medical College, Peshawar, Pakistan; ^5^ Public Health, Khyber Medical University, Peshawar, Pakistan; ^6^ Department of Biochemistry, Kabir Medical College, Peshawar, Pakistan; ^7^ Faculty of Dentistry, Jordan University of Science and Technology, Irbid, Jordan; ^8^ Faculty of Medicine, Jordan University of Science & Technology, Irbid, Jordan; ^9^ Department of Cardiology, Imperial College London, London, United Kingdom; ^10^ Department of Cardiology, National Heart and Lung Institute, Imperial College London, London, United Kingdom

**Keywords:** odontogenic tumors, mouth neoplasms, immune checkpoint inhibitors, molecular targeted therapy, OCS

## Abstract

Malignant odontogenic tumors are rare, accounting for only 1%–6.1% of all odontogenic tumors. Among them, odontogenic carcinosarcoma (OCS) is an exceptionally rare and aggressive malignant neoplasm originating from dental tissues. First recognized by the World Health Organization (WHO) in 1992, OCS is characterized by high-grade biphasic malignant epithelial and mesenchymal components, contributing to its aggressive clinical behavior. OCS often presents with nonspecific symptoms such as pain, swelling, and loosening of teeth, which complicate early diagnosis. Its rarity adds to the diagnostic challenges, frequently leading to delays in identification. Histopathological evaluation remains the cornerstone for accurate diagnosis, distinguishing OCS from other odontogenic tumors through features like epithelial nuclear pleomorphism, mitotic activity, and mesenchymal sarcomatous differentiation. Management typically involves surgical resection with clear margins, while adjuvant therapies such as chemotherapy and radiation are considered in select cases. Recent advancements in molecular oncology and surgical techniques, including robotic-assisted procedures and 3D-printed reconstructive aids, offer promising avenues for improving patient outcomes. A multidisciplinary approach and ongoing research are essential to enhance diagnostic accuracy, refine treatment protocols, and improve the prognosis for patients affected by this rare malignancy. The primary objective of this review is to consolidate current knowledge on OCS, focusing on its diagnostic complexities, treatment strategies, and potential emerging therapies.

## 1 Introduction

Malignant odontogenic tumors are rare, accounting for only 1%–6.1% of all odontogenic neoplasms, with odontogenic carcinosarcoma (OCS) being an exceptionally aggressive and uncommon subtype (1, 2). OCS, first officially recognized in the 1992 World Health Organization (WHO) classification of odontogenic tumors, poses significant diagnostic and therapeutic challenges due to its rarity, histological complexity, and high recurrence rates (2). The lack of standardized treatment protocols and limited molecular research further complicate clinical management, contributing to poor long-term outcomes.

OCS typically affects adults, with a mean age of presentation between 40 and 60 years, though cases have been reported from ages 20 to 80, with a slight male predominance (3). No significant racial or ethnic predisposition has been identified, and while etiological factors remain unclear, prior radiation exposure and genetic factors have been suggested as possible contributors (4). More than half of cases of OCS usually start in the posterior mandible, while maxillary cases are more aggressive because they may progress to the paranasal and base of the skull (1). Pain and paranesthesia are the main characteristics of clinical presentations, with many patients experiencing dental issues, such as loosened or missing teeth or dental implants, indicating severe disturbances in oral function (4). Lymphadenopathy, however, is relatively rare (3).

OCS is characterized by both malignant epithelial and mesenchymal components, either developing *de novo* or through sarcomatous transformation of benign odontogenic tumors like ameloblastoma or ameloblastic fibroma (5). Histologically, OCS is a biphasic tumor with diverse cellular components, including ameloblastic, clear cell, squamous, and spindle cells with varying degrees of myxoid to sclerotic differentiation [8]. The epithelial component displays nuclear pleomorphism, mitotic activity, cytokeratin positivity, basaloid or ameloblastic differentiation, and features reminiscent of squamous cell carcinoma (6). Meanwhile, the mesenchymal component exhibits sarcomatous characteristics, such as vimentin-positive spindle-shaped or pleomorphic cells (7). Transition zones, where epithelial and mesenchymal cells blend with a lack of cellular cohesion, are frequently observed (8).

Accurate diagnosis requires integrating clinical, radiographic, and histological evidence to distinguish OCS from similar tumors like squamous cell carcinoma, fibrosarcoma, ameloblastic carcinoma, and osteosarcoma, as well as from benign lesions such as dental cysts and fibro-osseous lesions or even metastatic tumors and Langerhans cell histiocytosis (1, 5, 6, 9). Genetic studies, though limited, indicate alterations in tumor suppressor genes such as *TP53* and dysregulation in pathways related to cell proliferation and apoptosis, potentially driving tumor aggressiveness and therapeutic resistance (1, 2).

Survival outcomes are highly variable, with a 5-year overall survival rate of 81% for malignant odontogenic tumors, though rates drop significantly with positive surgical margins (29% for involved margins vs. 78% for negative margins) (10, 11). Recurrence occurs in up to 30% of cases, and salvage surgery offers limited long-term success, with a 5-year survival of approximately 31% despite negative margins (12). Distant metastases, primarily to the lungs, lymph nodes, and bones, occur in 10%–15% of cases, further complicating prognosis (13).

Advanced imaging techniques, such as cone-beam computed tomography (CBCT) and magnetic resonance imaging (MRI), are essential for assessing tumor extent, while histopathological analysis remains the gold standard for diagnosis (6). Given the tumor's aggressive nature, radical surgical excision with clear margins is the cornerstone of treatment, though adjunctive systemic therapies (chemotherapy, radiation) are reserved for advanced or metastatic cases and lack robust evidence for efficacy (7). Emerging strategies, including robotic-assisted surgery and 3D printing, show promise, but further research is needed to refine treatment protocols and integrate molecular insights into clinical practice (9).

The objective of this study is to conduct a comprehensive literature review to consolidate current knowledge on OCS, focusing on clinical presentation, diagnostic complexities, therapeutic approaches, and emerging treatment strategies. This review aims to highlight gaps in existing literature and suggest directions for future research to improve patient outcomes.

## 2 Therapeutic approaches

**Figure 1:**
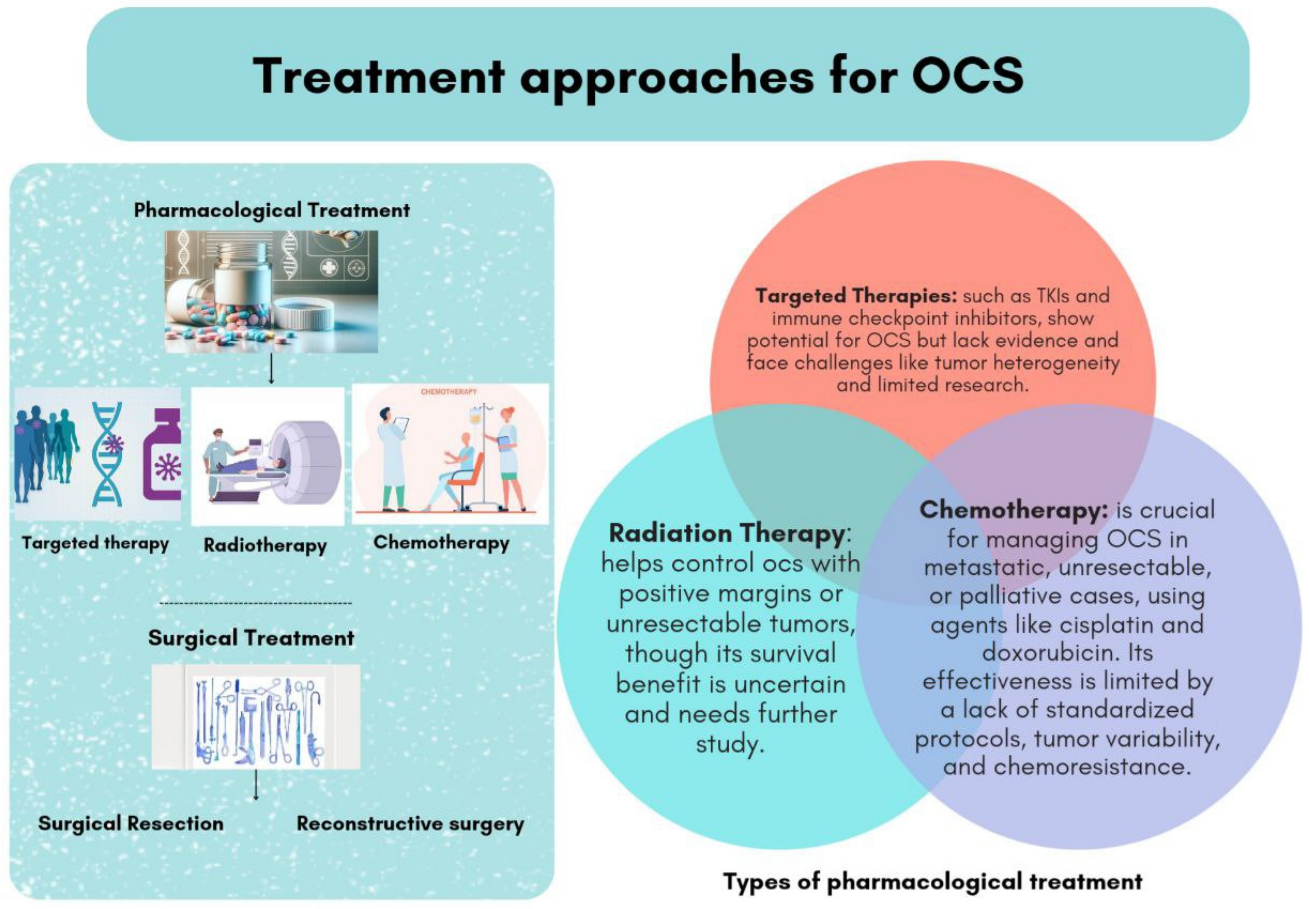
Types of pharmacological and non-pharmacological treatment for patients with odontogenic carcinosarcoma (OCS).

The management of OCS involves a multimodal approach, including surgical intervention, radiation therapy, and chemotherapy (Figure 1**)**.

### 2.1 Surgical treatment

#### 2.1.1 Surgical resection

Surgical resection remains the cornerstone of treatment for OCS, with the primary objective being the achievement of clear surgical margins. Local recurrence is a significant concern when margins are compromised, making complete tumor excision critical to optimizing patient outcomes (7, 9, 14). The extent of surgical intervention is determined by factors such as the size and location of the tumor and its involvement with surrounding anatomical structures (15).

En bloc resection involves the complete removal of the tumor along with a margin of healthy tissue. This technique is particularly valuable in minimizing the risk of local recurrence by ensuring that all cancerous cells are excised. It is especially preferred when the tumor invades critical structures, such as the mandible or maxilla, where precise surgical margins are essential for disease control (16).

For tumors localized to the mandible, segmental mandibulectomy may be performed. This procedure entails the resection of a specific segment of the mandible containing the tumor. However, this surgical approach often necessitates subsequent reconstructive efforts to restore function and aesthetics (15). Reconstructive techniques frequently employed include the use of free flaps or bone grafts, which help to restore the mandible and support masticatory function effectively (1, 6).

When the tumor involves the upper jaw, partial or total maxillectomy may be required. This procedure entails the removal of the maxilla, a surgery that can significantly affect oral function and facial appearance. Patients undergoing maxillary resection often face challenges related to speech, swallowing, and mastication, as well as aesthetic concerns due to changes in facial symmetry. Reconstructive options in these cases may include prosthetic devices or advanced surgical techniques to restore the contour of the face and the functional abilities of the oral cavity (14). Such interventions aim to address both functional and aesthetic needs, improving the quality of life for patients after extensive surgical resection.

#### 2.1.2 Reconstructive surgery

Reconstructive surgery plays a critical role following the extensive resections often necessary for OCS. These surgical interventions are essential for restoring both the form and function of the jaw, which are critical for normal speech, chewing, and aesthetic appearance (14). The main goal of reconstructive surgery is to ensure that patients regain functional abilities while maintaining a satisfactory aesthetic outcome. This aspect of care is vital, as resections for OCS often result in large structural defects (17).

Autogenous bone grafting is a widely used technique that involves harvesting bone from the patient's body, typically from the iliac crest or fibula. This approach is advantageous because autogenous bone is biocompatible and integrates seamlessly with existing bone structures, making it ideal for reconstructing defects created by tumor resection (15). Autogenous bone grafting not only provides structural support but also facilitates rapid healing and better long-term outcomes (18). For extensive defects, computer-assisted surgical planning has emerged as a useful tool for enhancing the precision and effectiveness of graft placement (19).

Microvascular free flap reconstruction is another advanced technique used in cases of larger or more complex defects. This approach involves transferring tissue, including skin, muscle, and bone, from a donor site to the jaw area while maintaining the tissue's blood supply through microvascular connections. The fibula is one of the most used donor sites due to its size, shape, and structural compatibility with the mandible (17). Other potential donor sites include the radial forearm and scapula, which are selected based on patient-specific needs (19). This technique allows for significant restoration of both aesthetic and functional aspects of the jaw, enabling patients to achieve better oral function and facial contour (20).

Reconstructive surgery is crucial for restoring essential functions such as masticatory efficiency and speech, which are vital for maintaining nutritional intake and quality of life (19). Many patients experience significant improvements in their ability to chew and speak following successful reconstructive procedures. Equally important are aesthetic considerations, as extensive resections often lead to facial asymmetry and deformities. Reconstructive techniques help restore facial symmetry and improve self-esteem, which can profoundly impact psychological well-being (14).

To achieve optimal outcomes, reconstructive surgery frequently involves a multidisciplinary approach, engaging oral and maxillofacial surgeons, plastic surgeons, and rehabilitation specialists. Such collaboration ensures that the functional and aesthetic needs of the patient are comprehensively addressed (20). Emerging technologies, such as virtual surgical planning and intraoperative navigation, have further improved precision in reconstructive efforts, contributing to better patient outcomes and recovery times (19).

### 2.2 Radiation therapy

Radiation therapy plays an important role as an adjuvant treatment in the management of OCS, particularly in cases where surgical margins are positive or when complete surgical resection is not feasible. Its primary goal is to reduce the risk of local recurrence by targeting residual tumor cells, thereby enhancing locoregional control. From other malignancies, patients with positive surgical margins, radiation therapy serves as a critical adjunct by addressing microscopic disease that might otherwise contribute to recurrence (21, 22). Similarly, in cases where the tumor is unresectable or close to critical anatomical structures, radiation therapy offers a means of disease control, alleviating symptoms and potentially improving survival outcomes (23).

Radiation therapy regimens typically involve delivering doses between 60 and 70 Gy in fractionated schedules over several weeks. This approach balances effective tumor control while minimizing damage to surrounding healthy tissue (23, 24). Evidence from related malignancies suggests that external beam radiation therapy (EBRT) alone or in combination with brachytherapy can significantly improve local control and reduce recurrence rates. For instance, EBRT has been associated with improved survival rates in uterine and other carcinosarcomas, supporting its role as an adjuvant treatment in aggressive malignancies like OCS (24, 25).

Despite its advantages in local control, the impact of radiation therapy on long-term survival for OCS remains less clear. While radiation therapy effectively manages residual disease, its ability to improve overall survival outcomes has been debated. Patient-specific factors, including tumor grade, histological features, and comorbidities, significantly influence treatment efficacy (26). Moreover, the combination of radiation therapy with other modalities, such as chemotherapy or targeted therapies, has shown synergistic effects in managing other aggressive carcinosarcomas, suggesting a potential avenue for improving outcomes in OCS (27, 28).

Innovative approaches, including stereotactic body radiation therapy (SBRT) and advanced imaging-guided delivery techniques, have improved precision in targeting tumors. These methods allow for higher doses to be delivered to the tumor while sparing adjacent tissues, enhancing therapeutic outcomes with fewer side effects (29). However, challenges persist, particularly in determining the optimal combination of radiation and systemic therapies to maximize efficacy without exacerbating toxicity. Ongoing research and clinical trials are needed to refine radiation protocols specifically for OCS and integrate them into multidisciplinary treatment plans (14, 30).

### 2.3 Chemotherapy

There are no standardized chemotherapy protocols for OCS due to its rarity. However, treatment approaches for related malignancies like ameloblastic carcinoma (AC) and head and neck sarcomas provide useful guidance. AC, a malignant odontogenic tumor with similar behavior to OCS, is often treated with chemotherapy regimens used for head and neck cancers, including cisplatin, doxorubicin, and cyclophosphamide. These agents have shown effectiveness, especially when surgery alone is insufficient (31). Additionally, chemotherapy regimens for head and neck sarcomas, typically including doxorubicin, ifosfamide, and cisplatin, have been used in clinical practice, with varying success. Though these protocols are mainly designed for soft tissue sarcomas, they have been considered for odontogenic sarcomas like OCS due to the tumor's aggressive nature (32). Case reports have suggested that regimens including cisplatin, doxorubicin, and cyclophosphamide may show some efficacy in OCS, especially in cases of recurrence or metastasis, though the evidence is limited (33). Therefore, while these chemotherapy protocols from related malignancies can serve as a foundation for OCS treatment, further research and clinical trials are needed to establish a standardized, evidence-based chemotherapy regimen for OCS.

The chemotherapeutic agents commonly used in OCS include cisplatin, doxorubicin, and cyclophosphamide. Cisplatin, a platinum-based agent, exerts its effect by forming DNA cross-links that prevent replication, ultimately leading to apoptosis (34). It is usually administered intravenously and often combined with other agents to enhance therapeutic outcomes. However, cisplatin is associated with significant side effects, including nephrotoxicity, nausea, and myelosuppression (35). Doxorubicin, an anthracycline antibiotic, works by intercalating into DNA and disrupting replication, inducing cell death. It is a key component of combination regimens for sarcomas but carries a risk of cardiotoxicity, alopecia, and immunosuppression (36). Cyclophosphamide, a DNA-alkylating agent, interferes with DNA replication and is typically used in multi-drug regimens. Its side effects include myelosuppression, nausea, and a potential increased risk of secondary malignancies (9). Combination regimens, such as cisplatin-doxorubicin or cisplatin with ifosfamide, are commonly considered to enhance outcomes, particularly for tumors with sarcomatous phenotypes. However, the efficacy of these combinations in OCS remains largely speculative due to the limited number of reported cases and lack of prospective studies (37).

Several documented case reports offer insights into the use of chemotherapy for OCS, highlighting both its potential benefits and limitations: In one case, a 56-year-old male with recurrent odontogenic carcinosarcoma of the mandible underwent surgical resection followed by adjuvant chemotherapy with cisplatin and doxorubicin. While the chemotherapy resulted in a partial response with a reduction in tumor size, the tumor recurred several months later, indicating that the treatment, although initially effective, was not sufficient for long-term control (38). In another case, a 42-year-old female diagnosed with odontogenic carcinosarcoma of the maxilla was treated with a combination of cyclophosphamide, doxorubicin, and cisplatin. This regimen led to a partial response and tumor shrinkage, but the patient experienced recurrence and metastasis, resulting in a relatively short survival time post-treatment (39). Similarly, a 70-year-old male with odontogenic carcinosarcoma of the mandible was treated with cisplatin as an adjuvant therapy after surgical resection. While the chemotherapy provided brief tumor control, the patient developed metastatic disease within a year and had local recurrence after 18 months, ultimately leading to a poor long-term prognosis (40). In a pediatric case, a 15-year-old child diagnosed with odontogenic carcinosarcoma involving both the jaw and soft tissues was treated with a combination of cisplatin, doxorubicin, and cyclophosphamide. This regimen led to initial tumor reduction, but recurrence was observed after nine months, necessitating further treatment with radiotherapy (41).

Despite its importance, the use of chemotherapy in OCS faces several challenges. The lack of established treatment protocols complicates clinical decision-making, and the scarcity of cases makes it difficult to conduct large-scale clinical trials to validate the efficacy of specific regimens (42). Additionally, the heterogeneity of OCS tumors, with both epithelial and mesenchymal components, contributes to variable responses to treatment, and some patients may exhibit intrinsic or acquired chemoresistance (43). Innovative approaches, such as dose-dense chemotherapy and nanoparticle-based drug delivery systems, are being explored to overcome these limitations and improve outcomes (44). These strategies aim to maximize tumor cytotoxicity while minimizing systemic side effects, but further research is required to establish their effectiveness in OCS (45).

### 2.4 Targeted therapy

Recent advances in molecular oncology have opened new avenues for the treatment of OCS through targeted therapies. These approaches aim to interfere with specific molecular pathways involved in tumor development and progression, offering potentially higher efficacy and reduced side effects compared to conventional chemotherapy (5).

Tyrosine kinase inhibitors (TKIs) represent a promising class of targeted agents. Erlotinib, an epidermal growth factor receptor (EGFR) inhibitor, blocks pathways critical to tumor survival and proliferation. It is widely used in cancers such as non-small cell lung cancer and pancreatic cancer and could be relevant in OCS cases with demonstrated EGFR overexpression (6). Sorafenib, a multi-kinase inhibitor targeting pathways such as *RAF-MEK-ERK*, has shown efficacy in inhibiting angiogenesis and tumor growth. While it is primarily approved for hepatocellular carcinoma and renal cell carcinoma, its application in OCS requires further investigation to determine clinical benefit (46). Despite these promising mechanisms, the use of TKIs in OCS remains largely speculative due to the paucity of evidence in this specific tumor type (5, 46).

Immune checkpoint inhibitors have emerged as a breakthrough in oncology, especially in tumors exhibiting immune evasion. Pembrolizumab, a PD-1 inhibitor, enhances the immune system's ability to recognize and destroy cancer cells. Its efficacy has been demonstrated in tumors with PD-L1 overexpression, making it a potential candidate for OCS cases with similar profiles (35). Nivolumab, another PD-1 inhibitor, functions similarly and has shown success in several solid tumors, including head and neck cancers (41, 47). Although these therapies show promise, their utility in OCS is not well-established, highlighting the need for dedicated clinical trials (41, 47). Immune checkpoint inhibitors (ICIs) such as pembrolizumab (PD-1 inhibitor) and nivolumab (PD-1 inhibitor) have demonstrated promising results in various cancers, especially those with immune evasion mechanisms. While odontogenic carcinosarcoma (OCS) has not been studied directly in clinical trials for ICI treatment, ongoing trials in related cancers such as head and neck squamous cell carcinoma (HNSCC) and sarcomas may provide valuable insights. In the case of HNSCC, numerous trials are investigating the efficacy of pembrolizumab and nivolumab for patients with advanced or metastatic disease, particularly those exhibiting high PD-L1 expression. For instance, the KEYNOTE-012 trial, which evaluated pembrolizumab in advanced HNSCC patients, showed that a subset of patients with high PD-L1 expression experienced clinical benefit, indicating that PD-1 inhibitors could potentially be effective in tumors like OCS, which may have similar immune evasion mechanisms (48). Furthermore, soft tissue sarcomas, which share mesenchymal features with OCS, have also been the subject of studies investigating ICIs like nivolumab and pembrolizumab. The SARC028 trial investigating nivolumab in various soft tissue sarcomas showed mixed results, but some subtypes demonstrated promising responses, suggesting that similar therapies could be effective in OCS (49).

In addition to ICIs, TKIs such as sorafenib and erlotinib are already approved for cancers like hepatocellular carcinoma (HCC), renal cell carcinoma (RCC), and non-small cell lung cancer (NSCLC). These agents are also being investigated in clinical trials for HNSCC and soft tissue sarcomas, and may offer useful insights for OCS treatment. For example, a Phase II trial of erlotinib, an EGFR inhibitor, is exploring its efficacy in patients with recurrent or metastatic HNSCC. Additionally, the EXTREME trial assessed the combination of cetuximab, another EGFR-targeting agent, with chemotherapy for advanced HNSCC, improving survival rates in patients with EGFR overexpression (50). These results highlight the potential for EGFR-targeted therapies in cancers like HNSCC and, potentially, OCS if EGFR overexpression is present. Moreover, sorafenib, a multi-kinase inhibitor, has shown efficacy in sarcomas, including undifferentiated pleomorphic sarcoma and angiosarcoma. Maki et al., trial investigating sorafenib in advanced sarcomas has highlighted its potential for reducing tumor size, particularly in sarcomas with vascularization mechanisms like those seen in OCS (51) This suggests that TKIs like sorafenib could be beneficial for treating OCS, particularly in cases where tumor vascularization is a critical factor. While OCS itself remains rare and there are no dedicated clinical trials for this malignancy, ongoing studies in related cancers provide essential insights into potential therapies for OCS. The use of ICIs and TKIs in tumors with immune evasion mechanisms or vascularization characteristics similar to those in OCS could offer promising therapeutic avenues.

Challenges remain in the implementation of targeted therapies for OCS. The rarity of this malignancy complicates the identification of actionable molecular targets and the conduct of large-scale clinical trials (52). Tumor heterogeneity, characterized by the biphasic nature of OCS, further adds to the complexity, as both epithelial and mesenchymal components may respond differently to targeted agents (52). Innovations such as next-generation sequencing and comprehensive genomic profiling are essential to uncover novel molecular pathways that could guide the use of targeted therapies in OCS (53).

Future directions include the exploration of combination regimens involving targeted therapies and conventional treatments like chemotherapy or radiation. For instance, combining EGFR inhibitors with immune checkpoint inhibitors could provide synergistic effects by simultaneously targeting tumor growth and enhancing the immune response (54). Advances in molecular diagnostics and biomarker identification will be pivotal in personalizing treatment strategies, optimizing outcomes, and minimizing adverse effects in OCS patients (52). A summary of the treatment modalities, including their indications and challenges, is provided in (Table 1**)** to enhance understanding of the therapeutic approaches discussed.

**Table 1:** Summary of treatment modalities for odontogenic carcinosarcoma.

Treatment option	Details	Indications	Challenges/limitations
Surgical treatment	– En bloc resection: Complete removal of the tumor with healthy margins (1, 6). – Segmental mandibulectomy or maxillectomy: Removal of affected bone segments with reconstruction if needed (14, 15)	– Primary management for localized disease. – When clear margins can be achieved	– Difficulty in achieving clear margins due to tumor proximity to critical structures. – Functional and aesthetic impairments post-surgery (14)
Reconstructive surgery	– Bone grafts (autogenous from iliac crest/fibula) or microvascular free flap reconstruction (17) – Custom implants using 3D printing for precision (19)	– To restore function and aesthetics after extensive resections	– Risk of graft failure (18) – Requires multidisciplinary expertise and advanced technology (20)
Chemotherapy	– Common agents: cisplatin, doxorubicin, cyclophosphamide (34–36). – Combination regimens: Cisplatin-doxorubicin or Cisplatin-Ifosfamide (37)	– Metastatic disease. – Incomplete surgical resection. – Palliative care for advanced stages	– Lack of standardized protocols (42). – Limited efficacy in managing chemo resistant tumors (43). – Significant side effects like nephrotoxicity and cardiotoxicity
Radiation therapy	– Doses of 60–70 Gy administered over multiple fractions (21). – Often used as an adjuvant therapy post-surgery (23)	– Positive surgical margins. – Cases where complete resection is not feasible	– May not significantly improve overall survival (26). – Risk of damage to surrounding healthy tissues (24, 25)
Targeted therapy	– Tyrosine kinase inhibitors (Erlotinib, Sorafenib): target signaling pathways like EGFR or MAPK (46). – Immune checkpoint inhibitors (Pembrolizumab, Nivolumab): enhance T-cell activity (47)	– Tumors with molecular abnormalities (e.g., EGFR overexpression or PD-L1 presence)	– Insufficient clinical evidence for routine use. – High cost and limited availability (52)

OCS, odontogenic carcinosarcoma; Gy, gray (unit of radiation dose); EGFR, epidermal growth factor receptor; MAPK, mitogen-activated protein kinase; PD-L1, programmed death-ligand 1; CAR-T, chimeric antigen receptor T-cell.

## 3 Complications and challenges in treatment

### 3.1 Diagnostic challenges

Diagnostic challenges further complicate management, as the rarity and nonspecific presentation of OCS often lead to delayed diagnosis. Symptoms such as pain, swelling, tooth mobility, and facial asymmetry can mimic benign odontogenic lesions, while conventional radiographs may reveal poorly defined radiolucent lesions, making differentiation from other malignancies difficult (55). The biphasic histopathology of OCS, with malignant epithelial and mesenchymal components, adds another layer of complexity, complicating differentiation from tumors like ameloblastic carcinoma and spindle cell carcinoma (56). To address these diagnostic hurdles, advanced imaging techniques such as CBCT and MRI are invaluable. CBCT excels at visualizing bony structures and surgical planning, while MRI is superior for assessing soft tissue involvement, and using both together provides a more comprehensive tumor assessment. Molecular diagnostics, including immunohistochemical markers like cytokeratin and vimentin, alongside next-generation sequencing (NGS), can enhance diagnostic precision and uncover potential therapeutic targets. Multidisciplinary tumor boards involving pathologists, radiologists, oncologists, and geneticists can further refine diagnostic accuracy and guide personalized treatment strategies.

### 3.2 Therapeutic challenges

Given the aggressive nature of the tumor and the need for extensive resections, postoperative complications are common. Infection risk is elevated due to the complexity of the surgical site, immunosuppression, and pre-existing health conditions. Prompt management with antibiotics, abscess drainage, or secondary surgical interventions is essential to prevent infection progression and ensure optimal recovery (14). Achieving negative surgical margins remains a critical challenge, especially in anatomically complex regions, as tumors near vital structures often preclude complete resection. Positive margins are associated with higher recurrence rates and poor prognosis, necessitating the use of adjuvant therapies, such as radiation or re-excision, to manage residual disease (57). Despite these interventions, complete disease eradication remains difficult due to the tumor's aggressive local invasion and high recurrence propensity.

Therapeutic management is equally complex due to the lack of standardized treatment protocols and the tumor's variable response to conventional therapies. Radical surgical resection with an emphasis on achieving clear margins remains the cornerstone of treatment, though reconstructive surgery is often necessary to restore function, particularly when patients experience functional impairments like speech, mastication, and swallowing difficulties post-surgery (58). Even with aggressive resection, recurrence is common due to factors such as tumor biology, positive margins, and inadequate adjuvant therapy (5). Personalized medicine, guided by molecular profiling, offers promising avenues, with targeted therapies (e.g., tyrosine kinase inhibitors, immune checkpoint inhibitors) showing potential for improved outcomes. Combining radiotherapy with novel systemic agents, such as immunotherapies and targeted drugs, may enhance treatment efficacy, particularly in recurrent or metastatic cases. Emerging modalities, including photodynamic and gene therapies, are being explored to improve local control and minimize systemic side effects.

Ultimately, addressing the complications and challenges associated with OCS requires a multifaceted, multidisciplinary approach. Continuous research, global collaboration, and advancements in diagnostic and therapeutic modalities will be pivotal in improving outcomes for patients facing this rare and aggressive malignancy.

## 4 Future directions

### 4.1 Advances in surgical techniques

Recent advancements in surgical techniques, particularly robotic-assisted surgery and 3D printing, offer promising potential to enhance outcomes in complex cases of odontogenic carcinosarcoma. Robotic-assisted surgery enables minimally invasive procedures with improved precision and control, facilitating access to challenging anatomical areas and allowing for more accurate tumor resections while minimizing damage to surrounding critical structures. This approach is associated with reduced postoperative pain, shorter recovery times, and fewer complications (59). 3D printing technology allows for the creation of patient-specific anatomical models based on preoperative imaging, aiding in surgical planning and enabling surgeons to visualize the tumor's relationship with surrounding tissues. This enhanced understanding can lead to more strategic surgical approaches, improving the chances of achieving clear margins and successful reconstruction (60). Additionally, 3D printing facilitates the fabrication of custom implants or guides tailored to the unique anatomical needs of each patient, enhancing the fit and functionality of reconstructive components and improving overall patient satisfaction and outcomes (61). The integration of these advanced techniques into clinical practice requires collaboration among surgeons, biomedical engineers, and imaging specialists to optimize surgical interventions. Furthermore, these technologies serve as valuable tools for training and simulation, allowing surgeons to practice complex procedures in a risk-free environment before performing them on patients (62).

### 4.2 Emerging therapeutic approaches

Emerging therapies offer hope for improving the treatment of OCS, a rare and aggressive malignancy. Gene therapy aims to target genetic mutations or mechanisms driving tumor growth but remains theoretical for OCS due to limited molecular profiling. Identifying consistent genetic alterations will be critical for advancing this approach. Similarly, CAR-T cell therapy, which has shown success in hematological cancers, faces challenges in OCS due to the immunosuppressive tumor microenvironment and the lack of tumor-specific antigens (63). Precision medicine tailors' interventions to the unique genomic profile of a patient's tumor, with actionable mutations like *BRAF V600E*, identified in related odontogenic carcinomas, serving as potential targets for therapy (64, 65).

Collaborative research, ongoing clinical trials, and a multidisciplinary approach involving oncologists, geneticists, and immunologists are essential for integrating these emerging treatments into clinical practice (66, 67). These efforts are crucial for addressing treatment challenges, improving patient outcomes, and advancing personalized therapeutic strategies.

## 5 Conclusion

OCS is an exceptionally rare, aggressive malignant neoplasm characterized by its biphasic histopathological features and challenging clinical presentation. The rarity of OCS contributes to significant diagnostic and therapeutic hurdles, often leading to delayed diagnoses and limited treatment options. Diagnostic challenges stem from nonspecific clinical symptoms, radiographic ambiguity, and overlapping histopathological features with other odontogenic and non-odontogenic neoplasms. Advanced imaging modalities, such as CBCT and MRI, molecular diagnostics, and multidisciplinary team involvement are essential to improve diagnostic accuracy.

Therapeutic management remains complex due to the lack of standardized treatment protocols, high recurrence rates, and resistance to conventional therapies such as chemotherapy and radiotherapy. Surgical resection with clear margins remains the cornerstone of treatment, supplemented by reconstructive techniques to restore function and aesthetics. Personalized treatment strategies, informed by molecular profiling, offer promising avenues, with targeted therapies and immunotherapies emerging as potential options. Despite these advances, the absence of evidence-based guidelines and the limited responsiveness to adjuvant therapies pose ongoing challenges.

Addressing these gaps requires a multidisciplinary approach, continuous research, and global collaboration to develop standardized treatment protocols. Future efforts should focus on comprehensive molecular profiling, innovative therapeutic modalities, and the integration of emerging technologies in surgical and diagnostic practices. Enhancing early diagnosis and refining therapeutic strategies will be critical in improving patient outcomes and survival rates for this rare and aggressive malignancy.
